# Cases of Surgery

**Published:** 1845-06

**Authors:** Daniel Brainard

**Affiliations:** Prof. of Surgery in the Rush Medical College


ILLINOIS

MEDICAL & SURGICAL JOURNAL.

VOL. II. JUNE, 1845. NO. 3.

*Cases of Surgery.* By DANIEL BRAINARD, M. D., Prof. of Surgery in the Rush Medical College.

CASE 1. *Strangulated Hernia. Operation. Recovery.*—May 28, 1845, I was called at 1 o'clock, A. M., to visit J. M. Q., aged about 35 years, laboring under a strangulated inguinal hernia of the left side. He gave the following history of the case:—For several years back he had been affected with reducible inguinal herniæ of both sides, for which he had worn a double truss; occasionally one or the other would slip down, but could be easily replaced. The evening previously, however, it came down on the left side and could not as usual be replaced, which he attributed to the state of obstinate costiveness in which his bowels had remained for several days. On seeing him I found him affected with great pain, retching, tenderness about the tumor, which was about 3 inches long by 2 broad. I immediately made attempts to reduce it by the *taxis*, but not succeeding administered small doses of Ant. Tart. and bled him copiously, until most perfect and prolongued syncope and prostration were induced; when placing his hips in an elevated position, flexing the thighs and relaxing the abdominal muscles, I made a persevering effort at reduction, but without success. Directing cold applications to be made to the tumor, and attempts to be made to procure discharges by stool, I then left him until morning, when I visited him in company with Drs. Kimberley, Brinckerhoff and Dyer. The patient was still in the same state; and after some additional but unsuccessful efforts at the *taxis*, I proceeded to the performance of the operation 14 hours after the occurrence of the accident. The patient having been long affected with varicocele, the tumor was covered with an unusual plexus of enlarged veins. On laying open the sac, a a knuckle of small intestine was seen at the upper part, while the principal portion was formed of omentum. On slightly enlarging the external ring, where there was some stricture, and pressing the finger to the internal ring, a still greater constriction was found at this latter, on the division of which the intestine was easily replaced. The omentum, however, was extensively adherent to the sac, and required considerable dissection to separate it. At length this was effected, and the entire contents reduced. Stitches and adhesive straps were applied, and a large part of the wound healed by the first intention. Purges and *lavements* were administered, and in a few hours copious stools were procured, all pain and irritation subsided—and at present, June 3, he is convalescent.

*Remarks.*—There are two points worthy of being particularly attended to in reference to this case. The first is, the safety and success of the operation when seasonably performed—an observation which has been often made, but which cannot be too often repeated. The second is in relation to the seat of the stricture, a point which has been of late much agitated, without however being settled. In the present case, it was evident that the greatest degree of constriction existed at the internal ring.

Case II. *Spontaneous Gangrene of the Thumb. Amputation. Recovery.*—Mrs. L., æt. 50 years, presented herself, May 30, 1845, having a necrosis of the bone of the extreme phalanx of the thumb of the right hand. The following was her account of the case:—Six weeks previously she had been attacked with pain in the end of the thumb, and of the fore and middle fingers, which increased until a small brown spot appeared upon the end of the thumb; this enlarged until it occupied the whole extremity of it, when it became circumscribed, and separated, leaving the extremity of the bone exposed and necrosed. On examination the necrosis was found to extend to the whole bone. Amputation at the articulation was performed, and in a week the patient returned home, the wound having healed by the first intention.

No cause of the gangrene could, on enquiry, be discovered.

As no treatment was employed to arrest its progress, it seems to have appeared and ceased spontaneously.

